# Strontium-90 activity concentration in soil samples from the exclusion zone of the Fukushima daiichi nuclear power plant

**DOI:** 10.1038/srep23925

**Published:** 2016-04-06

**Authors:** Sarata Kumar Sahoo, Norbert Kavasi, Atsuyuki Sorimachi, Hideki Arae, Shinji Tokonami, Jerzy Wojciech Mietelski, Edyta Łokas, Satoshi Yoshida

**Affiliations:** 1 Project for Environmental Dynamics and Radiation Effects, National Institute of Radiological Sciences, 4-9-1 Anagawa, Inage-ku, Chiba 263-8555, Japan; 2 Department of Radiation Physics and Chemistry, Fukushima Medical University, 1 Hikarigaoka, Fukushima, Fukushima 960-1295, Japan; 3 Institute of Radiation Emergency Medicine, Hirosaki University, 66-1 Hon-cho, Hirosaki City, Aomori 036-8564, Japan; 4 Department of Nuclear Physical Chemistry, Institute of Nuclear Physics, Polish Academy of Sciences, Krakow, Radzikowskiego 152, Poland

## Abstract

The radioactive fission product ^90^Sr has a long biological half-life (˜18 y) in the human body. Due to its chemical similarity to calcium it accumulates in bones and irradiates the bone marrow, causing its high radio-toxicity. Assessing ^90^Sr is therefore extremely important in case of a nuclear disaster. In this work 16 soil samples were collected from the exclusion zone (<30 km) of the earthquake-damaged Fukushima Daiichi nuclear power plant, to measure ^90^Sr activity concentration using liquid scintillation counting. ^137^Cs activity concentration was also measured with gamma-spectroscopy in order to investigate correlation with ^90^Sr. The ^90^Sr activity concentrations ranged from 3.0 ± 0.3 to 23.3 ± 1.5 Bq kg^−1^ while the ^137^Cs from 0.7 ± 0.1 to 110.8 ± 0.3 kBq kg^−1^. The fact that radioactive contamination originated from the Fukushima nuclear accident was obvious due to the presence of ^134^Cs. However, ^90^Sr contamination was not confirmed in all samples although detectable amounts of ^90^Sr can be expected in Japanese soils, as a background, stemming from global fallout due to the atmospheric nuclear weapon tests. Correlation analysis between ^90^Sr and ^137^Cs activity concentrations provides a potentially powerful tool to discriminate background ^90^Sr level from its Fukushima contribution.

Radioactive strontium isotopes in the atomic mass number range of 89 to 92 are generated with high cumulative fission yield (5–6%) during thermal neutron fission in a nuclear reactor^1^,^2^. Nuclear accidents can cause radioactive contamination of the environment, as it happened when the Fukushima Daiichi Nuclear Power Plant (FDNPP) was severely damaged by earthquake and tsunami on 11 March 2011. Such event can include radioactive strontium isotopes to disperse into the environment. In this situation ^91^Sr and ^92^Sr have little radiological significance owing to their short physical half-life (a few hours) but this is different for ^89^Sr (half-life 51 days) and ^90^Sr (29 years). Nuclear accidents are not the only source of ^89^Sr and ^90^Sr isotopes: atmospheric nuclear weapon tests^3^, misconducted underground nuclear weapon tests^4^, improper handling of by-products of nuclear weapon production^5^,^6^ or normal operation of nuclear facilities (e.g. reprocessing plants)^3^ can also result in contamination. As far as known only ^90^Sr was present in the environment of Japan before the Fukushima accident, derived from global fallout from large-scale bomb tests conducted from 1945^7^,^8^ and perhaps from the Chernobyl accident in 1986, the latter however a very small contribution, in comparison, if any. ^89^Sr has decayed since, thus its presence is an indicator of recent contamination. However, one year after emission by a nuclear event, ^89^Sr cannot be determined in environmental samples owing to its short half-life, thus the identification of the origin of ^90^Sr could be a challenging task. In our case, it consists of discrimination between Fukushima-borne and global ^90^Sr after decay of ^89^Sr.

Compared to volatile radioisotopes of caesium released into the atmosphere (^137^Cs, ˜15 PBq), non-volatile ^90^Sr (˜0.14 PBq) emission has been estimated two orders lower. ^90^Sr contamination is also expected in environmental samples due to Fukushima accident^9^. Moreover, ˜80% of the ^90^Sr emission was deposited over the Pacific Ocean, significantly decreasing the terrestrial pollution^10^, whereas no ^90^Sr concentration that would be of radiological concern (i.e. over 100 Bq kg^−1^) in soil samples have been reported^11^,^12^ outside the damaged FDNPP by the authorities.

In June 2011, Ministry of Education, Culture, Sports, Science and Technology (MEXT) and Nuclear Regulation Authority (NRA) Japan conducted a radiation survey that included ^89^Sr and ^90^Sr analysis in the vicinity of the damaged reactors of FDNPP in Fukushima prefecture^12^. Figure 1 shows the radiostrontium concentration levels in sampling points with detection of ^89^Sr and ^90^Sr over 10 Bq kg^−1^ along north-west direction. It was reported that ^89^Sr and ^90^Sr has been detected in 45% and 80% of samples, respectively, with average background level of ^90^Sr about 3 Bq kg^−1^ (n = 100)^12^,^13^.

Gamma emitter radionuclides such as iodine, tellurium and caesium isotopes^14^,^15^,^16^,^17^,^18^,^19^,^20^ have been published and studied much more extensively after the Fukushima accident than the pure beta emitter radiostrontium isotopes^21^,^22^,^23^,^24^. The explanation of this phenomenon is in the measurement technique: it is much simpler to measure gamma than pure beta emitters. Gamma rays are emitted in discrete energies, so qualitative identification of radioisotopes can be achieved easily by analysing the characteristic of energy spectra of gamma rays without element specific separation and complex sample preparation^25^. On the contrary, the energy distribution of the emitted electrons in the beta decay is continuous, therefore element specific separation from the interfering beta emitters is necessary for qualitative radioisotopes identification and subsequent measurement, making ^90^Sr determination complicated, time-consuming procedure with corrosive and aggressive chemicals during sample preparation^1^,^26^.

To increase the database of ^90^Sr, a new separation laboratory was established in the National Institute of Radiological Sciences, Japan (NIRS) in 2012. Sixteen soil samples were collected from the exclusion zone in the north-western direction of the FDNPP in 2013 to determine ^90^Sr and radiocaesium concentrations. The measurements of ^90^Sr concentration were duplicated, at the laboratories of NIRS and the Department of Nuclear Physical Chemistry, Institute of Nuclear Physics, Polish Academy of Sciences, Poland (IFJ-PAN).

Attempts were made to establish a procedure to distinguish ^90^Sr contamination caused by the Fukushima accident and atmospheric nuclear weapon tests by analysing the frequency distribution of the ^90^Sr activity concentration and obtaining a correlation of ^90^Sr and ^137^Cs. Finally, the measured data were compared to other data obtained by MEXT, NRA and other scientific research laboratories.

## Results

**Figure 2:**
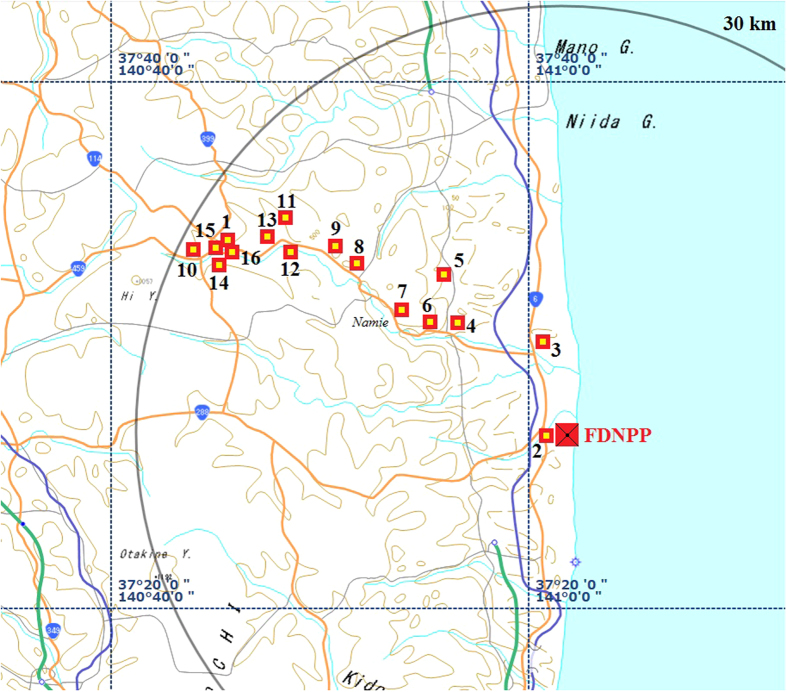
Locations of sampling points in the exclusion zone of FDNPP. Map source: Geospatial Information Authority of Japan (GSI) http://maps.gsi.go.jp/#10/37.357059/140.787048. The original map was modified using Adobe Photoshop Elements 9 software.

In our study 16 top layer (up to a 10 cm depth) soil grab samples were collected from the north-western direction of the FDNPP (Namie district) in the exclusion zone (<30 km from the FDNPP). This area was chosen because the highest radiation doses have been reported in this district due to wet deposition of radionuclides from the radioactive plumes released on 15 March 2011^19^,^20^,^27^. Locations and other details of the sampling points are shown in Fig. 2 13 and Table 1. The ^90^Sr and radiocaesium data of seven points (No. 1–4 and No. 7–9) were recently published elsewhere as preliminary results of this study^21^,^22^ and discussed here again to establish an extended database on ^90^Sr. A little contaminated area (No. 3), along north direction, was also monitored to compare with background ^90^Sr concentration.

Fukushima radioactive contamination was confirmed by the presence of ^134^Cs in all collected samples. The ^134^Cs/^137^Cs activity ratio was 1.0 at the time of the accident^17^. In this study, the average value of this ratio was the same, 1.0 (SD 0.02, n = 16) (decay correction date: 15 March 2011).

The results of the activity concentrations of ^90^Sr and ^137^Cs in this study are presented in Table 1 and as it can be seen, no cases of ^90^Sr concentrations exceeding 100 Bq kg^−1^ were detected. As expected, lowest activity concentrations of ^90^Sr and ^137^Cs were found at the little contaminated site (No. 3). ^90^Sr activity above 20 Bq kg^−1^ was revealed in two locations, No. 2 and No. 9, respectively. No. 2 was the closest sampling point to the FDNPP with the second highest ^137^Cs activity (97.9 ± 0.3 kBq kg^−1^), however No. 9 was a distant point over 20 km with somewhat lower ^137^Cs activity (58.2 ± 0.2 kBq kg^−1^).

### Frequency distribution

**Figure 3:**
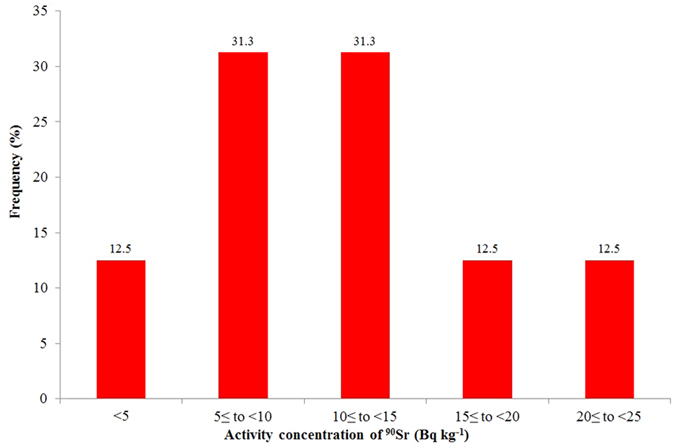
Frequency distribution of ^90^Sr in soil samples collected from the exclusion zone of FDNPP in 2013 (n = 16).

Figure 3 shows the frequency distribution of ^90^Sr activity concentration in the 16 analysed soil samples. The ^90^Sr activity concentrations were below 5 Bq kg^−1^ in 12.5% of samples analysed while in the major part, 62.5% were between 5 and 15 Bq kg^−1^ and above 15 Bq kg^−1^, 12.5% were detected.

### Correlation

**Figure 4:**
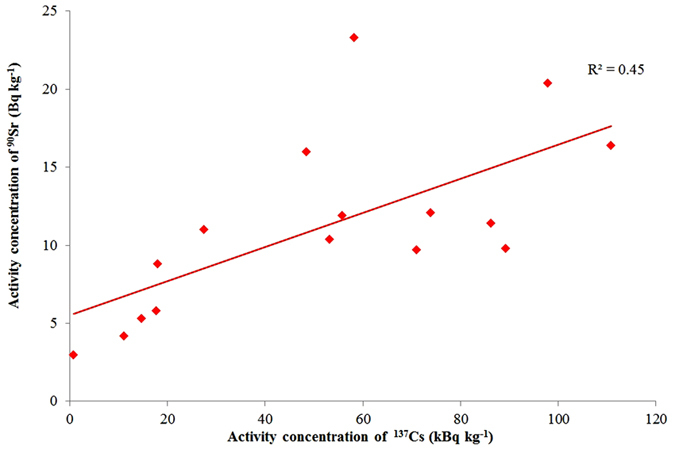
Correlation between ^90^Sr and ^137^Cs activity concentration in soil samples collected from the exclusion zone of FDNPP in 2013 (n = 16).

The correlation between the ^90^Sr and ^137^Cs concentration in the soil samples collected from the exclusion zone of the FDNPP is moderate as can be seen in Fig. 4. Thus ^137^Cs concentration (or ambient gamma dose rate) does not predict ^90^Sr concentration accurately. The activity concentrations of ^90^Sr were very similar (˜11 Bq kg^−1^) at sampling points No. 4, 7, 8, 13 and 15 but ^137^Cs concentrations fluctuated between 27.4 ± 0.1 and 86.2 ± 0.3 kBq kg^−1^ at these sites.

## Discussion

In 2005, i.e. before the Fukushima accident, MEXT had conducted a radiation monitoring survey at 55 sampling sites around Fukushima prefecture^11^. Low level ^90^Sr contamination likely originating from atmospheric nuclear weapons tests with an average of 3.0 Bq kg^−1^ were reported, with minimum and maximum <0.2 and 17.7 Bq kg^−1^, respectively. The maximum value of this baseline study shows that ^90^Sr concentrations of 20 Bq kg^−1^ cannot confirm contamination from Fukushima if the site specific background is not known exactly. The same 55 sampling points were also monitored in 2011 after the Fukushima nuclear accident and a slight increment of ^90^Sr level was found^11^. The reported average was 5.6 Bq kg^−1^ with minimum and maximum <0.9 and 80.8 Bq kg^−1^, respectively. The high maximum value found in this study clearly points to Fukushima contamination. Comparing the values detected before and after the Fukushima accident using Z score test (k = 2), a significant increase was found at 54.5% of the 55 sampling points, although the increment was marginal (<3 Bq kg^−1^) in most cases (83.3%). Significant increment was not verified at the maximum background point (17.7 Bq kg^−1^). (Decay correction date of this database was June to July 2011. Correction to 15 March 2011 was not possible hence the exact date was not published but the estimated decay could be around 1%, which is negligible).

**Figure 5:**
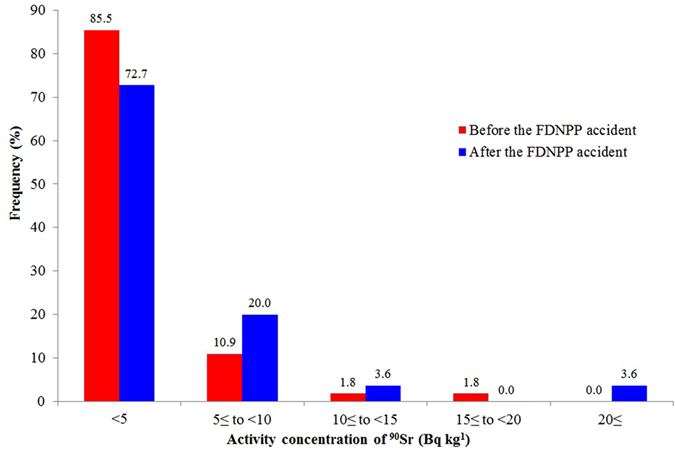
Frequency distribution of ^90^Sr in Fukushima prefecture before (2005) and after (2011) Fukushima accident (n = 55).

Figure 5 presents the frequency distributions of ^90^Sr concentrations measured before (2005) and after (2011) the FDNPP accident. The frequency distribution before the accident denotes that the dominating background level is below 5 Bq kg^−1^ (85.5%), while about 11% of the measured values were between 5 and 10 Bq kg^−1^ and about 4% were between 10 and 20 Bq kg^−1^.

Analysing the frequency distribution of the detected ^90^Sr values after the accident, slight increments can be seen in the ranges above 5 Bq kg^−1^. However, the dominating ^90^Sr level remained below 5 Bq kg^−1^ (72.7%). The frequencies were duplicated in the range of 5 to 10 Bq kg^−1^ and above 10 Bq kg^−1^. The differences in the frequency distribution before and after the accident verify ^90^Sr contamination from the Fukushima accident in Fukushima prefecture. Relatively low decrement (˜10%) in the range below 5 Bq kg^−1^ indicates that the ^90^Sr dispersion over the Fukushima prefecture was limited and the major part of the atmosphere released ^90^Sr was deposited in the proximity of the FDNPP. The theory of limited ^90^Sr dispersion was also supported by result of two-sample Kolmogorov-Smirnov statistical test. The cumulative distributions of the before and after results did not show significant difference; p value was 0.06 which is greater than 0.05 that is commonly considered as threshold of significance.

In our study, information about background ^90^Sr is not available. In order to decide whether a contribution of Fukushima ^90^Sr is present, we performed two analyses, one based on frequency distribution analysis of the measured ^90^Sr concentrations, the second on correlation between ^90^Sr and ^137^Cs.

### Comparison of frequency distribution

The database of our study was small (n = 16) as compared to MEXT’s (n = 55), and the sample collection was focused on one specific area instead of the whole prefecture. We assume that the frequency distribution of pre-Fukushima (background) ^90^Sr in our samples is similar to the one in MEXT’s study conducted in 2005, although the geographical areas of their origins are different (entire Fukushima prefecture for the old vs. Namie district for the new samples). Comparing Fig. 5, red bars (samples before Fukushima), with Fig. 3 (our samples), very different shapes of the distributions were noticed. The distribution of our samples is symmetric, centred around 5–15 Bq kg^−1^ while the background samples are positively skewed, concentrated in the class <5 Bq kg^−1^. Comparing the cumulative distribution of the two datasets by two-sample Kolmogorov-Smirnov statistical test significant difference was proved; p value was 1.6 × 10^−7^, i.e. highly significant at 0.05 level.

The results of the frequency distribution analysis emphasize the importance of environmental baseline studies in countries with nuclear energy reactors or other nuclear facilities.

### Analysis of correlation coefficients

The second method to identify Fukushima contribution to observed ^90^Sr concentration relies on correlation with other quantities. In sampling radiometric survey, the simplest way to reveal significant radioactive contamination is measuring ambient dose rate, which is produced by the gamma-rays emitted by the deposited radiocaesium isotopes (after the decay of iodine and tellurium isotopes). However, due to the different physical and chemical properties of alkali metals (Cs) and alkali earth metals (Sr), the presence of radiocaesium contamination cannot quantify ^90^Sr contamination with precision. The ratio between ^137^Cs and ^90^Sr in the fallout may not be preserved in the samples because of different environmental behaviours of the elements. The ratio observed in the samples is therefore different from the one in fallout, in general. The degree of difference is in addition different between sites (i.e. between soil types). Therefore, the correlation suffers not only because of the different behaviours of Sr and Cs, but additionally, because even these differences are not constant between sample points.

**Figure 6:**
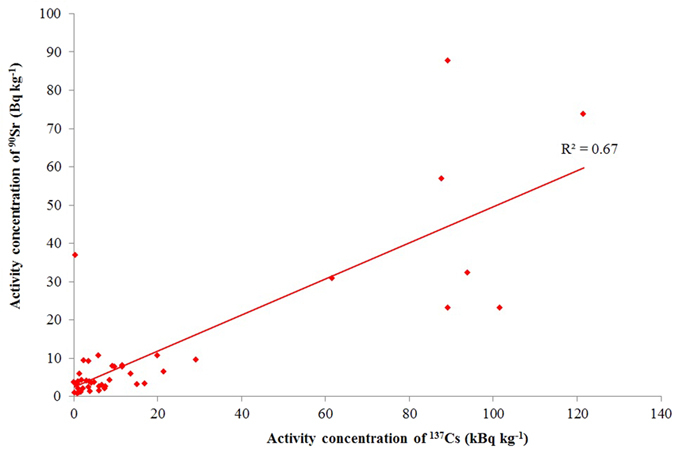
Correlation between ^90^Sr and ^137^Cs activity concentration in Fukushima soil samples collected in 2011 where the presence of ^89^Sr was confirmed (n = 45).

In spite of this fact, the data analysis of the MEXT and NRA’s database^12^ demonstrates that the correlation between the ^137^Cs and ^90^Sr deposition rate was strong in June 2011 after the accident as can be seen in Fig. 6. According to correlation coefficient of linear regression analysis (R^2^), about 70% of the ^90^Sr deposition is predicted from the ^137^Cs deposition at each sampling point in the vicinity of the FDNPP wherein ^90^Sr contamination from Fukushima was confirmed by the detection of ^89^Sr.

**Figure 7:**
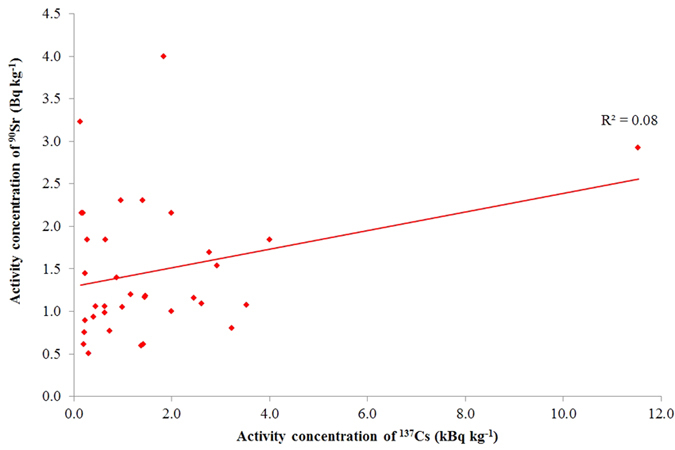
Correlation between ^90^Sr and ^137^Cs activity concentration in Fukushima soil samples collected in 2011 where the presence of ^89^Sr was not confirmed (n = 35).

Figure 7 shows the correlation between ^137^Cs and ^90^Sr where ^89^Sr was not detected, which means that ^90^Sr contamination by the Fukushima accident was very small, if present at all. One can see visually that correlation has practically disappeared and the ^90^Sr in these samples can be assumed to be almost exclusively pre-Fukushima ^90^Sr. As a consequence, can be concluded that ^137^Cs (or gamma dose rate) indicates the presence of ^90^Sr, although prediction is uncertain because of the imperfect correlation.

**Figure 8:**
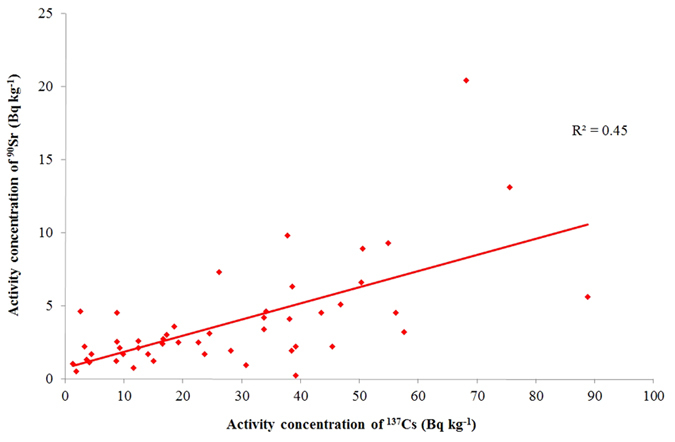
Correlation between ^90^Sr and ^137^Cs activity concentration in Fukushima soil samples collected in 2005 (n = 48).

On the other hand, in the presence of background contamination only of both ^137^Cs and ^90^Sr, as is the case in the pre-Fukushima samples of 2005, one can observe a moderate correlation (R^2^ = 0.45), Fig. 8. Presence of ^134^Cs may be due to recent contamination by FDNPP than global fallout and was not detected in these samples.

In our study, the samples were collected approximately two years after the Fukushima accident. The R^2^ was 0.45 (Fig. 4) which is lower than the R^2^ (0.67) calculated from database in which the Fukushima ^90^Sr contamination was confirmed by the presence of ^89^Sr (Fig. 6). Reasons for the lower R^2^ may be seen in the smaller number of samples, the longer time elapsed between fallout and sampling which gave more time for ecological processes which distort the original ratio, and a higher contribution of ^90^Sr pre-Fukushima background. However, the R^2^ is high enough to reject the null hypothesis, there is no correlation, with p < 0.005 error probability, which confirms the presence of Fukushima ^90^Sr contamination in the monitored area.

There was a good agreement between the database of our ^90^Sr results with MEXT and NRA’s database^12^. In the MEXT and NRA’s database, noticeable ^90^Sr concentrations (>˜5 kBq kg^−1^) and ^89^Sr concentrations could not be determined if the ^137^Cs level was below ˜12 kBq kg^−1^. In the database of our study, ^90^Sr concentrations below 5 Bq kg^−1^ were related to ˜15 kBq kg^−1^ or lower ^137^Cs concentrations.

Table 2 shows ^90^Sr results in soil samples from various references, including, in some cases, sampling depth. The soil samples were collected from different depths between 2.5 cm and 10 cm and on different dates from 30 March 2011 to 02 July 2012. In case of nuclear accidents, the depth of sampling is one of the most important factors to understand migration of radionuclides due to contamination. Most of the deposited ^90^Sr could be found on the soil surface (1–2 cm) shortly after the accident. A few years later it could be expected to have migrated into deeper layers (˜10 cm) due to different redistribution processes in soil. The velocity of the migration is site-specific and particularly depends on soil type, the amount of precipitation and permeability^28^,^29^. Therefore, 74 Bq kg^−1^ of ^90^Sr measured in 2.5 cm soil layer directly after the accident (No. 3) could have migrated into deeper layers during the following years^29^. Assuming ^90^Sr concentration migration velocity is a few cm per year, we can obtain a value for ^90^Sr about 20 Bq kg^−1^ approximately up to 10 cm depth. Accepting this theoretical approach, our results are in good agreement with the result of Maekawa *et al.* (No. 1 and 2), Takagai *et al.* (No. 3–5), and Mishra *et al.* (No. 6–9)^24^,^30^,^31^. On the other hand, Steinhauser *et al.*^23^ (No. 10–18) have reported very high ^90^Sr concentrations (up to 1,000 Bq kg^−1^) as well as extremely high ^137^Cs concentration (up to 4,600 kBq kg^−1^). The possible explanation of the high contamination could be that soil samples were collected from very thin surface layers (organic horizons) at highly contaminated spots within 4 km of FDNPP accident, even though the sampling was carried out after six months of the accident.

Also in Table 2, quite high radionuclide depositions were reported from the north-western direction of the FDNPP which is not surprising because this is the direction of the main contamination trace; however noticeable ^90^Sr contaminations (˜20 and ˜270 Bq kg^−1^) were also detected in the south direction (No. 8, 9 and 15) about 15 km from FDNPP^23^,^24^. These results emphasize the importance of radionuclides monitoring beyond the Namie district. It has been recognized that the ^137^Cs contamination due to Fukushima was quite low (˜1 kBq kg^−1^) at these southern points (No. 8 and 9) giving a contradiction to our theory stated above that considerable ^90^Sr contamination is not expected if the ^137^Cs contamination level is around 10 kBq kg^−1^ or lower. However, this contradiction could be accepted as result of rarely occurring anomaly as it can be observed in Fig. 8 where ^90^Sr concentrations above 20 Bq kg^−1^ are related to ^137^Cs below 100 Bq kg^−1^ but further study is necessary to prove this theory.

One more discrepancy can be noticed in results of Maekawa *et al.*, samples No 1 and 2 in Table 2. Considering the fact that date of the ambient gamma dose rate measurement was shortly (a few weeks difference) after the accidental radionuclides were released (12–31 March 2011) and the radioactive contamination was considerable (^137^Cs concentration was 262 kBq kg^−1^), the measured ambient radiation dose rate was quite low (˜20 μSv h^−1^) at these points. During the accident many other volatile and gamma-ray emitter radionuclides (iodine and tellurium isotopes) with shorter physical half-life (max. 33.6 d) were also released into the atmosphere and deposited in the terrestrial environment generating extremely high dose rates in the early days of the accident in the exclusion zone^32^, therefore much higher dose rate should have been measured than the reported ˜20 μSv h^−1^ in 18 April 2011. Higher dose rate (˜70 μSv h^−1^) was measured (No 6 and 7) with lower ^137^Cs concentration by Mishra *et al.*^24^, and similar dose rate was determined in our study after two years of the accident (Table 1, No 2).

## Methods

### Sample collection and preparation

The detailed information about sample collection and preparation for gamma spectroscopy measurement are given elsewhere^21^,^33^. In brief, at each sampling point, five grab samples (up to 10 cm depth) were collected from an area of about 1 m^2^, of which four from four vertices and one from the centre point. The five samples were mixed and placed in a plastic bag. During sample collection, the ambient gamma radiation dose rate was measured on a triplicate basis at a height of 1 m using a Radeye PRD-ER (Thermo Scientific, USA) personal radiation detector (with a micro-photomultiplier NaI(Tl)-detector). In the laboratory, the soil samples were dried at 105 °C for 24 hours. The dried soil samples were homogenized and sieved (<2 mm), stones and plant remains were removed. After this procedure, U8 standard cylindrical containers were filled up to 1–3 cm height with the soil samples to measure the specific activity of radiocaesium isotopes for 1800 sec.

### Reagents and materials

Tamapure AA-100 ultrapure hydrochloric, hydrofluoric, nitric and perchloric acids were used for the mass spectrometric measurement. Wako high pure hydrochloric, hydrofluoric and nitric acids were used for preparing the radiometric measurement. Standard solutions were prepared using high-purity water (>18 MΩ cm^−1^) produced with the Milli-Q2^TM^ water purification system. The detailed procedure for the separation of strontium is discussed elsewhere^22^,^34^. To summarize, the chemically digested soil samples (10–15 g) were dissolved in 8M HNO_3_ to ensure the highest strontium retention on Sr resin. These solutions were loaded into L-size polypropylene gravity columns (the flow rate was 1–2 ml min^−1^) attached with 50 ml syringes. The columns were packed with approximately 3 g (˜10 ml) Sr extraction chromatography resin (part number SR-B50-A) of 100–150 μm particle size (Eichrom Technologies, Inc., USA). The loaded Sr resins were first washed with 8M HNO_3_ and then with 3M HNO_3_ + 0.05M oxalic acid. Strontium was stripped from the column with 0.05M HNO_3_. The recovered amount of ^90^Sr was measured by a liquid scintillation counter. The chemical yields of strontium separations were between 25% and 70%.

### Instruments

The instruments used are described elsewhere^21^,^33^,^34^. Briefly, for strontium recovery calculation, stable strontium (^88^Sr) was analysed applying inductively coupled plasma mass spectrometer Agilent 8800 (Agilent Technologies, USA) at NIRS and ^85^Sr spike solution was used at IFJ-PAN. Gamma-spectrometer ORTEC GEM-100210 (ORTEC, USA) was used mounted with high-purity germanium detector and multichannel analyser for radiocesium determination. After the separation procedure, liquid scintillation counter TriCarb-3100 and Wallac 1414-003 (Perkin Elmer, USA) was used with Ultima Gold AB scintillation cocktail for ^90^Sr determination. The full analytical procedure was verified using soil reference sample produced by the International Atomic Energy Agency (IAEA 375), and tested using water samples in a world-wide open proficiency test, the IAEA-TEL-2014-03. The accuracy and precision of the applied procedure received accepted status.

### Statistical analysis

OriginPro 9 data analysis and graphing software (OriginLab Corp., USA) was used for running two-sample Kolmogorov-Smirnov statistical test.

## Additional Information

**How to cite this article**: Sahoo, S. K. *et al.* Strontium-90 activity concentration in soil samples from the exclusion zone of the Fukushima daiichi nuclear power plant. *Sci. Rep.* **6**, 23925; doi: 10.1038/srep23925 (2016).

## Figures and Tables

**Figure 1:**
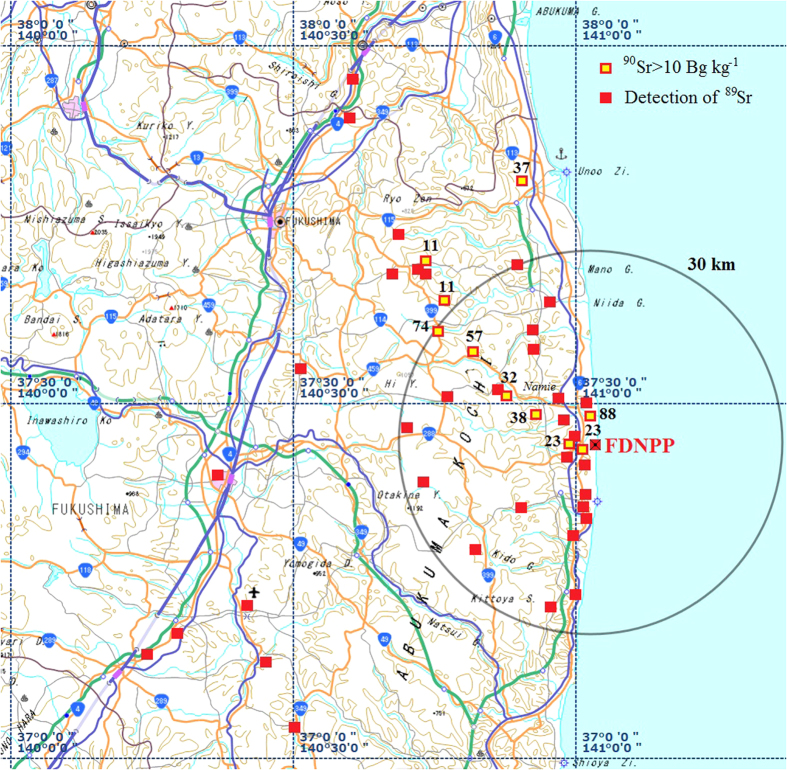
^89^Sr and ^90^Sr detection in soil samples collected in 2011 after FDNPP accident. Map source: Geospatial Information Authority of Japan (GSI) http://maps.gsi.go.jp/#10/37.357059/140.787048. The original map was modified using Adobe Photoshop Elements 9 software.

**Table 1:** Parameters and measurement results of the sampling points in the exclusion zone of the FDNPP.

No.	Sampling point		Distance from FDNPP km	Ambient gamma dose rate μSv h^−1^	Description of Sampling Site	Sampling date	^90^Sr Bq kg^−1^	^137^Cs kBq kg^−1^
	N	E						
1	37.558°	140.754°	28.1	6.5	grass field gravel	16 May 2013	9.7 ± 0.6	70.9 ± 0.7
2	37.423°	141.011°	1.9	21.9	forest	16 May 2013	20.4 ± 1.3	97.9 ± 0.3
3	37.497°	141.006°	8.0	0.6	grass field	16 May 2013	3.0 ± 0.3	0.7 ± 0.1
4	37.508°	140.932°	12.4	7.4	river bank grass	16 May 2013	11.9 ± 0.7	55.8 ± 0.2
5	37.528°	140.936°	13.8	4.5	grass field	16 May 2013	5.3 ± 0.4	14.7 ± 0.1
6	37.504°	140.914°	13.3	10.8	forest	16 May 2013	5.8 ± 0.4	17.6 ± 0.1
7	37.508°	140.901°	14.6	9.8	public garden	16 May 2013	10.4 ± 0.7	53.2 ± 0.2
8	37.542°	140.862°	19.6	16.9	grass field gravel	16 May 2013	11.0 ± 0.7	27.4 ± 0.1
9	37.553°	140.835°	22.0	17.4	forest	16 May 2013	23.3 ± 1.5	58.2 ± 0.2
10	37.555°	140.737°	29.3	2.9	agricultural field	17 May 2013	4.2 ± 0.3	11.1 ± 0.1
11	37.571°	140.794°	26.1	11.2	dam soil grass	17 May 2013	8.8 ± 0.6	17.9 ± 0.1
12	37.567°	140.799°	25.4	9.9	forest	17 May 2013	16.0 ± 1.1	48.4 ± 0.2
13	37.561°	140.792°	25.6	11.3	agricultural field	17 May 2013	11.4 ± 0.7	86.2 ± 0.3
14	37.553°	140.750°	28.2	3.4	forest	17 May 2013	16.4 ± 1.1	110.8 ± 0.3
15	37.558°	140.750°	28.6	5.8	forest	17 May 2013	12.1 ± 0.8	73.9 ± 0.3
16	37.558°	140.755°	28.2	6.4	grass field gravel	17 May 2013	9.8 ± 0.6	89.2 ± 0.3

Decay correction date: 15 March 2011.

**Table 2:** ^90^Sr and ^137^Cs activity concentration in Fukushima soil samples and parameters of sampling points by other researchers.

No.	Sampling date	Latitude N Longitude E	Sampling	Direction and distance from FDNPP [km]	Ambient gamma dose rate [μSv h^−1^]	^90^Sr conc. [Bq kg^−1^]	^137^Cs Conc. [kBq kg^−1^]	Reference
1	18 April 2011	–	Upper 5 cm	North–west, <30	19	58.6 ± 1.0	262*	31
2	20 April 2011	–	Upper 5 cm	South <20	5	10.3 ± 0.4	42.5*	31
3	30 March 2011	37.54399°	Upper 2.5 cm	North–west, 20	48.4	74.0*	–	30
		140.85481°						
4	29 August 2011	37.45946°	Upper 2.5 cm	North–west, 10	40	68.6*	–	30
		140.94378°						
5	30 August 2011	37.45946°	Upper 2.5 cm	North–west, 10	40	52.1*	–	30
		140.94378°						
6	24 November 2011	37.4140°	Upper 10 cm	North–west, <5	71	18.9 ± 3.5	62.2 ± 0.9	24
		141.0020°						
7	24 November 2011	37.4240°	Upper 10 cm	North–west, <5	85	8.6 ± 1.6	61.8 ± 0.9	24
		141.0004°						
8	23 November 2011	37.1982°	Upper 10 cm	South, <15	0.50	21.8 ± 2.6	1.0 ± 0.1	24
		140.9598°						
9	24 November 2011	37.1683°	Upper 10 cm	South, <15	0.45	22.9 ± 1.6	0.9 ± 0.1	24
		140.9894°						
10	21 December 2011	37.41716°	–	In front, 0.88	–	1070 ± 7.5	1790 ± 24.0	23
		141.02471°						
11	21 December 2011	37.41775°	–	In front, 1.5	–	303 ± 2.8	4600 ± 8.3	23
		141.01682°						
12	21 December 2011	37.41764°	–	In front, 1.9	–	31.8 ± 0.4	127 ± 0.5	23
		141.01225°						
13	21 December 2011	37.38874°	–	South-south west, 4.3	–	232 ± 2	2740 ± 11	23
		141.00831°						
14	21 December 2011	37.49574°	–	North-west, 8.7	–	29.7 ± 0.4	25 ± 0.3	23
		141.00137°						
15	21 December 2011	37.31489°	–	South, 12.0	–	268 ± 2.3	21.7 ± 0.2	23
		141.01416°						
6	21 December 2011	37.56588°	–	North-north west, 16.4	–	67 ± 0.7	62.2 ± 0.5	23
		140.99203°						
17	21 December 2011	37.56588°	–	North-north west, 16.4	–	8.9 ± 0.1	9.2 ± 0.1	23
		140.99203°						
18	20 July 2012	38.269°	–	North, 95	–	<3	5 ± 0.3	23
		140.869°						

Decay correction date: 15 March 2011 except data published by Takagai *et al.*^30^.

^*^uncertainty was not given.
